# Climatic controls on surface albedo and mass balance of Urumqi Glacier No. 1 in the last decade, 2014–2024

**DOI:** 10.1016/j.isci.2025.113942

**Published:** 2025-11-03

**Authors:** Hu Yuhan, Wang Feiteng, Zhang Chunxiang, Pan Ruiqi, Du Zhencai, Ming Jing

**Affiliations:** 1 College of Science, Shihezi University, Shihezi 832000, China; 2 Key Laboratory of Cryospheric Science and Frozen Soil Engineering, Northwest Institute of Eco-Environment and Resources, Chinese Academy of Sciences, Lanzhou 730000, China; 3 Transport Planning and Research Institute, Ministry of Transport, Beijing 100028, China; 4 Institute of Atmospheric Physics, Chinese Academy of Sciences, Beijing 100029, China; 5 Beacon Science & Consulting, Urrbrae, SA 5064, Australia

**Keywords:** earth sciences, climatology, earth-surface processes, glacial processes

## Abstract

Glaciers in Central Asia are retreating; we analyze how climate controls surface albedo on Urumqi Glacier No. 1 (UG1) using Landsat-8/9 (2014–2024) and ERA5-Land datasets integrated with the Google Earth Engine platform. Multiple linear regression and generalized additive models (GAMs) relate albedo to air temperature, snowfall, and aerosols. UG1’s albedo declines significantly (−0.006 years^−1^), concurrent with sustained mass loss (−0.019 m w.e. yr^−1^). Air temperature and snowfall are dominant controls: linear models explain 34.6% of variance, while GAMs increase explained variance to 45.7% and reveal nonlinear threshold behavior near 0°C. Seasonal skill varies widely (7.7–37.7%). Integrating 45 mass-balance stakes shows spatially heterogeneous albedo–mass-balance correlations (−0.5 to +1.0); glacier-wide correlation is weak (r = −0.075, *p* = 0.191), with positive correlations in upper zones consistent with temperature–albedo feedback. Roughly 54% of variance remains unexplained, highlighting roles for non-meteorological processes. Results support nonlinear parameterizations of albedo in glacier mass-balance models and improve future projections for a warming Central Asia.

## Highlights

• UG1 albedo declines by 0.006 per year (2014–2024; Landsat analysis)
• Glacier mass loss persists at −0.019 m w.e. per year over 2000–2024
• GAMs beat linear models (45.7% vs. 34.6%) and reveal 0°C thresholds
• Albedo–mass balance is spatially heterogeneous; 54% variance unexplained

## Graphical abstract

**Figure:**
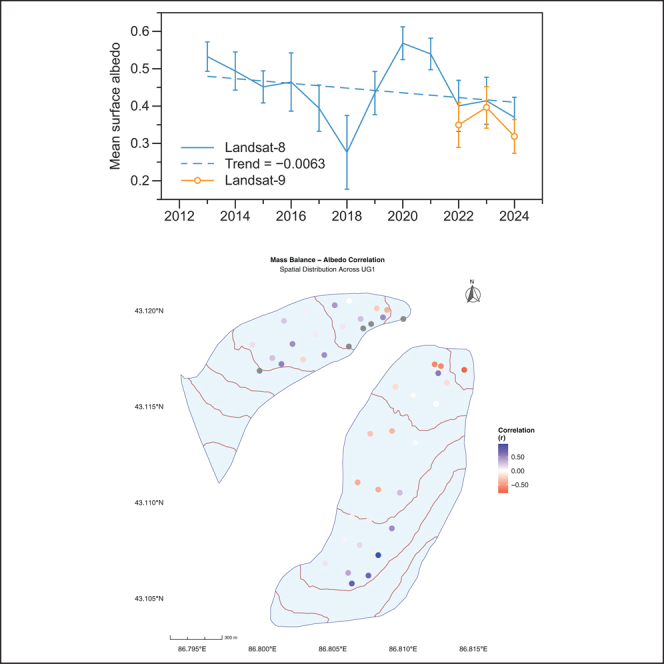

## Introduction

**Figure 1:**
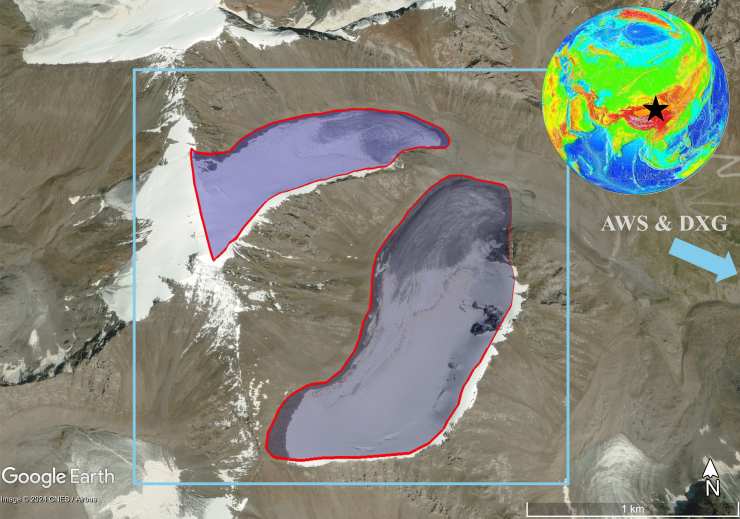
Outlined UG1 with red lines and blue shades in a satellite image on the Google Earth platform and its position in the globe (https://www.ngdc.noaa.gov/mgg/image/2minrelief.html) 2-m air temperature and precipitation were computed within the gray blue rectangle area from the ERA5-land dataset, and the Daxigou Meteorological Station (DXG) is indicated nearby.

Glaciers are vital components of global and regional hydrological systems, functioning as natural reservoirs that regulate water availability through seasonal melting.^1^^,^^2^ In arid regions like Central Asia, glacier meltwater sustains rivers critical for agriculture, domestic use, and ecosystem stability.^3^^,^^4^ Urumqi Glacier No. 1 (UG1), situated in the eastern Tianshan Mountains of Xinjiang, China, exemplifies this role and has been a focal point of glaciological research due to its sensitivity to climate change and its importance to downstream water resources (Figure 1).^5^ Over recent decades, UG1 has experienced accelerated retreat, a trend consistent with global glacier decline driven by rising temperatures.^6^^,^^7^ This retreat raises pressing concerns about the long-term sustainability of water supplies in the region.^8^^,^^9^

Surface albedo, the proportion of solar radiation reflected by a glacier, plays a pivotal role in regulating its energy balance and mass loss.^10^ Observational evidence indicates a declining albedo trend at UG1 during multiple non-consecutive periods of the past two decades (2000–2020), particularly during the ablation season, which has amplified melt rates.^11^^,^^12^^,^^13^ This decline aligns with broader patterns observed across High Mountain Asia, where warming climates have intensified glacier wastage.^14^^,^^15^

Air temperature and precipitation stands out as the primary climatic driver of albedo variation and glacier melt at UG1.^16^^,^^17^^,^^18^ In winter and spring, snowfall can delay the onset of melting by maintaining a bright surface. However, during the summer ablation season, precipitation in the form of snow is rare, and rainfall may even darken the glacier surface, further reducing albedo.^19^ The complex interactions between these meteorological variables and surface albedo require detailed quantitative analysis to understand their relative contributions to glacier evolution. Variability in these climate factors, influenced by regional climate dynamics, contributes to seasonal and interannual albedo fluctuations.^20^^,^^21^

This study examines albedo trends at UG1 from 2014 to 2024, focusing on the roles of air temperature and precipitation as key climatic drivers. By integrating satellite-derived albedo data with ground-based meteorological records, we analyze seasonal and long-term variations during the critical summer melt season. Our objective is to quantify how these factors shape albedo and, consequently, glacier mass balance in a warming climate. Such insights are crucial for projecting UG1’s future evolution and assessing the hydrological implications for Central Asia’s arid landscapes.^22^^,^^23^

## Results

### Variations of surface air temperature and precipitation over UG1

**Figure 2:**
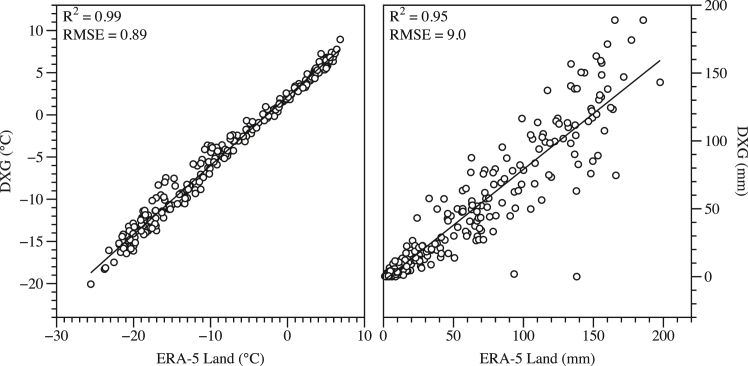
Scatterplots of monthly mean temperature and monthly sum precipitation by DXG and ERA5-Land

The analysis of monthly mean temperature and precipitation datasets, sourced from ERA5-Land reanalysis and automatic weather station (AWS) observations at the Daxigou Meteorological Station (DXG) site, reveals consistent and coherent climate characteristics at UG1. ERA5-Land and AWS datasets exhibited exceptionally strong correlations, with Pearson correlation coefficients of approximately 0.99 for temperature and 0.95 for precipitation (Figure 2). These high correlation values underscore the reliability of ERA5-Land data, despite minor systematic deviations when compared to ground measurements, as evidenced by statistically significant differences detected through paired *t* tests (*p* value <0.0001 for both temperature and precipitation).

**Figure 3:**
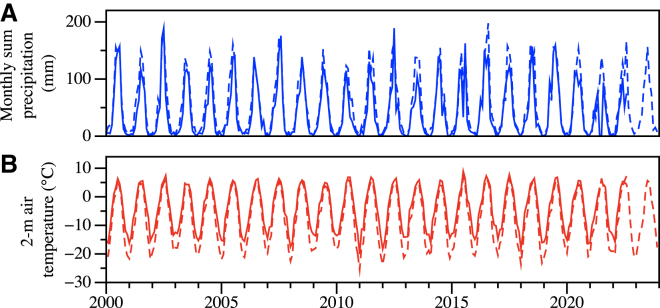
Precipitation and 2m air temperature over the study area Monthly total precipitation (A) and monthly mean 2-m air temperature (B) at UG1 from 2000 to 2020s. Dashed lines represent data derived from the ERA5-Land reanalysis dataset, while solid lines indicate direct measurements from the AWS at DXG. The comparison highlights close agreement between reanalysis and observed data, with subtle systematic deviations, reflecting slight warming and wetting trends over the two decades.

Seasonal analyses highlighted distinct annual cycles characterized by starkly cold winters and moderately warm summers, typical of high-altitude glacier environments. Temperature time series decomposition confirmed pronounced seasonality, showcasing substantial winter cooling and summer warming cycles (Figure 3). Notably, the clear seasonal precipitation cycles align with regional climatic patterns, reflecting the influence of synoptic-scale weather systems prevalent in the Tianshan Mountains region.

Furthermore, long-term trend analyses conducted via linear regression indicated subtle yet discernible climate changes over the period studied. The temperature exhibited a gradual warming trend, marked by an increase rate of approximately 0.019°C per year. Concurrently, precipitation trends revealed a slight but consistent increase, estimated at approximately 0.181 mm per year. These trends suggest incremental climatic shifts toward a slightly warmer and wetter regime, potentially affecting the glacial mass balance and hydrological dynamics at UG1.

### Mass balances of UG1

**Figure 4:**
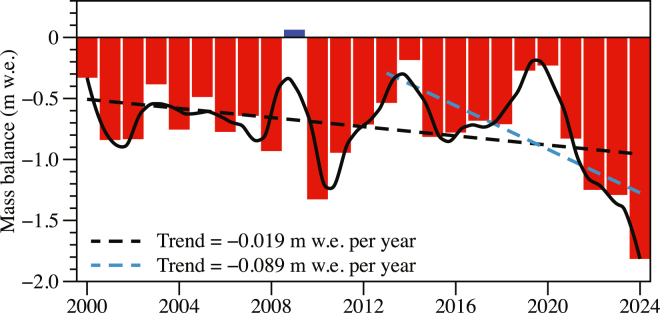
Annual mass balance of UG1 from 2000 to 2024, expressed in m w.e. Red bars represent annual glacier-wide mass balance values, with a single positive year shown in blue. The solid black line depicts a LOESS smoothing curve, illustrating interannual variability and short-term fluctuations. The dashed lines indicate linear regression trends, revealing persistent long-term mass loss at a rate of −0.019 m w.e. per year from 2000 to 2024 and −0.089 m w.e. per year from 2014 to 2024, respectively.

Figure 4 shows the annual glacier-wide mass balance of UG1 from 2000 to 2024, expressed in meters water equivalent (m w.e.).^24^ The mass balance values display substantial interannual variability, with consistently negative values in most years, indicating sustained mass loss. To characterize both the long-term trend and short-term fluctuations, a linear regression and a LOESS (locally estimated scatterplot smoothing) curve were applied. The LOESS fit captures the nonlinear variability in mass balance, reflecting the influence of interannual climate variability and episodic meteorological extremes. In contrast, the linear trend reveals a persistent negative trajectory, with a rate of change estimated at −0.019 m w.e. per year from 2000 to 2024 and even more at −0.089 w.e. per year from 2014 to 2024. This trend indicates a significant long-term thinning of UG1 over the past two decades, consistent with regional climatic warming and observed glacier retreat throughout the Tianshan region.

### UG1’s surface albedo

**Figure 5:**
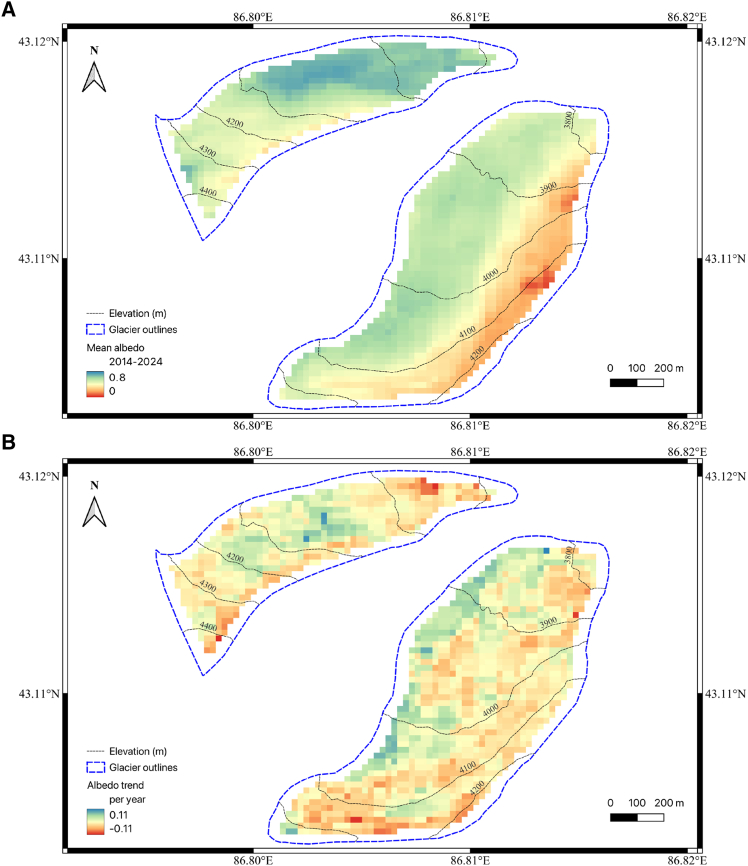
Mean albedo map and trend slopes on pixcel level for UG1 Spatial distribution of (A) mean surface albedo (2014–2024) and (B) albedo trends per year across UG1 (USGS Landsat-8 Level 2, Collection 2, Tier 1). Blue dashed lines represent glacier outlines, and black solid lines show elevation contours at 100-m intervals.

Figure 5 provides comprehensive insights into both the spatial distribution of surface albedo and its temporal evolution across UG1 based on Landsat-8 dataset, although the lack of a long *in situ* albedo series and the implication for uncertainty. The mean albedo distribution (Figure 5A) reveals complex spatial patterns that diverge from simple elevation-dependent relationships. Both glacier branches display greenish colors (moderate albedo values) at their lower elevations, with the eastern branch showing distinct spatial heterogeneity where reddish colors (lower albedo values) are primarily concentrated along the eastern and southern margins while central areas maintain higher albedo values. Most notably, the western branch exhibits an inverse elevation-albedo relationship, where higher elevations display lower albedo values (orange to red colors), contrary to typical expectations of increased reflectance with elevation.

The albedo trend analysis (Figure 5B) reveals that these spatial patterns of mean albedo do not directly correspond to patterns of temporal change. In the eastern branch, central and lower elevation areas predominantly display positive or neutral albedo trends (greenish colors), while negative trends (reddish colors) are concentrated along the margins, particularly the eastern and southern edges. The western branch exhibits a more complex pattern with mixed positive and negative trends distributed throughout different elevation zones, where areas showing strong negative trends appear scattered across both high and low elevations rather than following systematic elevation gradients. The western branch’s inverse elevation-albedo relationship reflects local topographic effects including enhanced wind exposure and snow redistribution at higher elevations, demonstrating that complex terrain geometry can override typical elevation gradients in albedo distribution.

**Figure 6:**
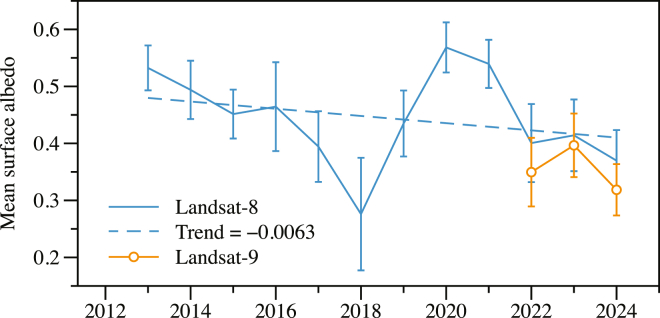
Temporal evolution of mean surface albedo across UG1 from 2013 to 2024 Blue line with error bars shows Landsat-8 observations, while orange line represents Landsat-9 data. The blue dashed line indicates the overall declining trend (−0.0063 per year) calculated from Landsat-8 data. Error bars represent standard error of the mean albedo values across all glacier pixels for each observation period.

The temporal evolution of mean surface albedo presented in Figure 6 demonstrates a consistent and statistically significant declining trend across UG1 over the past decade. The Landsat-8 record reveals a systematic decrease in surface reflectance at a linear rate of −0.0063 per year, representing a substantial change in the glacier’s radiative properties. This declining trend persists despite considerable interannual variability, suggesting that long-term climate forcing dominates over short-term meteorological fluctuations.

## Discussion

### Modeling albedo variation with a multiple linear model

**Figure 7:**
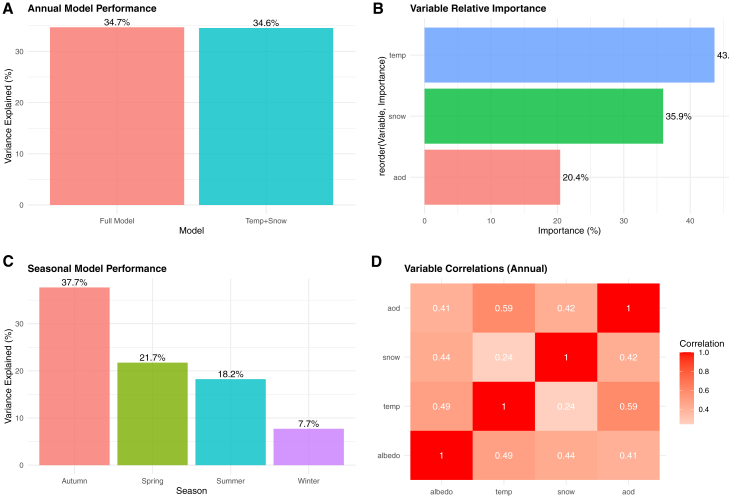
Climatic and environmental drivers of albedo variation at UG1 (A) Annual model performance comparison showing negligible improvement from AOD. (B) Relative importance of predictor variables with snowfall dominance. (C) Seasonal variation in model explanatory power ranging from 7.7% to 37.7%. (D) Variable correlation matrix revealing complex inter-relationships and substantial unexplained variance.

We assume surface air temperature, snow fall amount, and aerosol optical depth (AOD) as climatic and other factors primarily determining glacier albedo variation.^25^ Our linear modeling approach included two predictive models: a temperature-snowfall model (albedo = 0.448 + 0.009 × temperature + 0.004 × snowfall) and a full model incorporating AOD (albedo = 0.403 + 0.008 × temperature + 0.004 × snowfall + 0.285 × AOD). These linear models explained 34.7% of albedo variance, with the full model showing only negligible improvement (0.1%) over the temperature-snowfall model (34.6%) (Figure 7A).

Air temperature is the most important factor explaining 43% of the total explained variance by the model, following by snowfall (36%) and AOD (20%) (Figure 7B). The observed AOD–albedo association primarily reflects the radiative effect of atmospheric aerosols on incoming solar radiation; we do not infer surface darkening by aerosol deposition because deposition was not measured.

The relatively modest explanatory power of our models (34.7%) indicates that nearly two-thirds of albedo variation remains unexplained by the meteorological factors examined, highlighting the complex, non-linear nature of glacier albedo processes and the significant influence of non-meteorological factors. A previous study on the nearby UG1 has demonstrated that glacier mass balance shows very high sensitivity to albedo-related parameters, particularly snow albedo and the timescale determining how long snow albedo approaches firn albedo after snowfall.^26^

Our analysis reveals substantial seasonal variation in temperature-albedo relationships and model performance, with correlations ranging from negative to positive across different seasons and explained variance varying dramatically from 7.7% in winter to 37.7% in autumn (Figure 7C). The seasonal models demonstrate that the relative importance of temperature, snowfall, and aerosols changes throughout the year, with autumn showing the strongest predictive capability. This seasonal dependency reflects the complex interplay between temperature and surface conditions throughout the annual cycle. Studies have shown that warming on glaciers not only prolongs the melting period but also causes significant decreases in snow accumulation and additional decreases in surface albedo due to diminished summer snowfall.^27^ The positive temperature-albedo correlation observed in some seasons (Figure 7D) may result from temperature-dependent precipitation phase changes, where warmer conditions within the snowfall range promote fresh snow accumulation, thereby increasing albedo. Conversely, negative correlations likely reflect enhanced melting and ice exposure under warmer conditions.

### Modeling albedo variation with a generalized additive model

**Figure 8:**
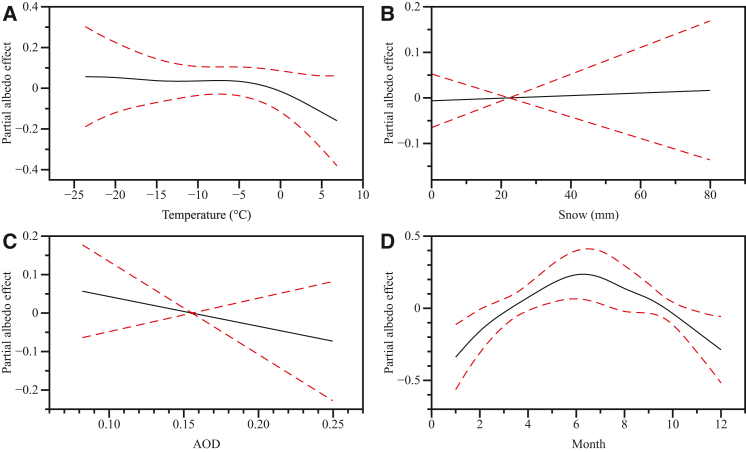
Nonlinear relationships between glacier albedo and environmental factors from GAM analysis at UG1 Partial effects plots show (A) temperature effects revealing complex threshold behaviors, (B) snow coverage effects demonstrating positive relationship with diminishing returns, (C) atmospheric optical depth effects indicating moderate atmospheric influences, and (D) seasonal patterns capturing strong cyclical variations. Shaded areas represent 95% confidence intervals.

To address the complex, non-linear nature of glacier albedo processes, we employed more flexible generalized additive models (GAMs) to capture potential non-linear relationships between albedo and environmental factors. The GAMs successfully captured complex nonlinear relationships between glacier albedo and environmental factors at UG1, explaining 46% of observed variance (Figure 8). Temperature effects (Figure 8A) revealed a critical threshold response at 0°C, with stable albedo values below freezing but pronounced decreases above 0°C as temperature increased. Snowfall amount demonstrated the expected positive relationship with albedo (Figure 8B), with snowfall events rapidly increasing surface reflectivity by covering dark ice surfaces, exhibiting steepest gains at low snow depths and diminishing returns at higher accumulations, confirming snow-albedo feedback as a key process in glacier energy balance. AOD (Figure 8C) showed moderate non-linear effects across the observed range (0.10–0.25), indicating that regional aerosol loading from dust transport in the arid Tianshan region influences both direct solar radiations reaching the surface and the accuracy of satellite-based albedo retrievals, with implications for remote sensing applications in dust-prone environments. Seasonal patterns (Figure 8D) dominated albedo variability throughout the year, displaying strong cyclical behavior with winter maxima during snow accumulation periods, summer minima during intensive melting, and rapid transitions during spring onset and autumn freeze-up, reflecting the overwhelming importance of annual climate cycles. These nonlinear relationships demonstrate that traditional linear parameterizations inadequately represent glacier albedo processes, emphasizing the need for sophisticated modeling approaches to accurately project glacier responses under future climate scenarios in high-mountain regions.

**Figure 9:**
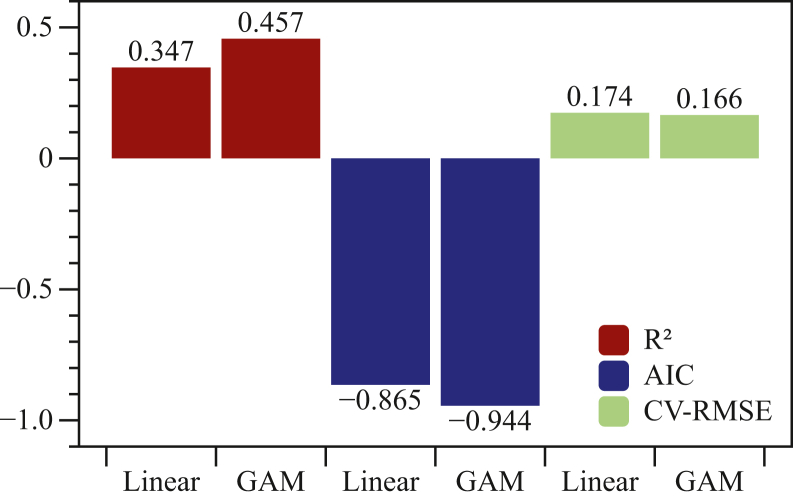
The comparison of R^2^ for explained deviance, AIC for model’s balance, and CV-RMSE for model’s accuracy between best linear and GAM models

We compared the modeling results between GAMs and multiple linear models. GAMs substantially outperformed traditional linear models, with the best-performing GAMs achieving an R^2^ of 0.457 compared to 0.347 for linear models—representing a 4.8% improvement in predictive accuracy (coefficient of variation of root mean squared error [CV-RMSE]: 0.166 vs. 0.174) (Figure 9). The GAM models’ superior performance metrics (better balanced Akaike information criterion [AIC], R^2^, and cross-validation RMSE) combined with their increased flexibility (11.6 effective degrees of freedom vs. 4 for linear models) indicate that glacier albedo responses to climate forcing exhibit nonlinear dynamics that require more sophisticated modeling approaches to properly characterize the complex interactions governing this critical component of glacier mass balance.

### Surface mass balance linked to high-resolution albedo

**Figure 10:**
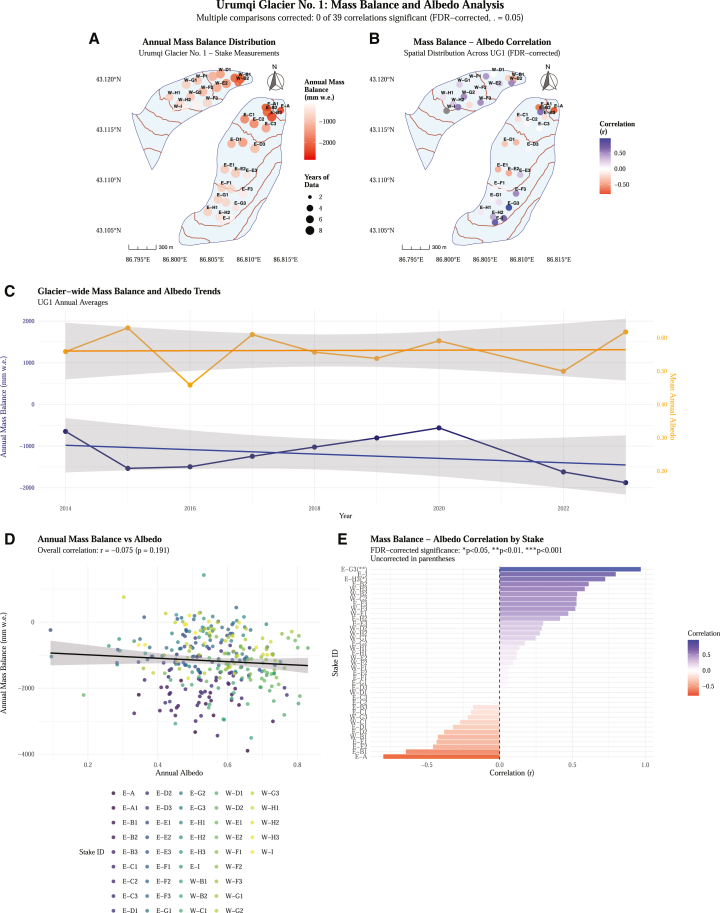
Comprehensive analysis of mass balance and albedo relationships across UG1 (A) Spatial distribution of annual mass balance at 45 stakes with ≥3 years of data (2013–2023), showing glacier outline (blue line) and elevation contours (brown lines). Point size indicates years of data availability (4–10 years). (B) Spatial pattern of mass balance-albedo correlations, with significance indicators (∗*p* < 0.05, ∗∗*p* < 0.01, ∗∗∗*p* < 0.001) shown as text labels on stake points. (C) Glacier-wide temporal trends showing annual mass balance (blue line, left axis) and mean albedo (orange line, right axis) with confidence intervals. The gray ribbon represents ±1 SD across all stakes. (D) Scatterplot of annual mass balance versus albedo for all stake-year combinations (*n* = 309), colored by stake ID. The overall correlation is weak and nonsignificant (r = −0.075, *p* = 0.191). (E) Individual stake correlations ranked from most negative to most positive, with significance levels indicated. Stakes E-H3 and E-G3 show statistically significant positive correlations.

The integration of high-resolution Landsat-derived albedo data with *in situ* mass balance measurements reveals complex spatial and temporal relationships across UG1 (Figure 10). Our analysis of 45 stakes with at least three years of concurrent data (2013–2023) provides insights into the albedo-mass balance feedback mechanisms operating on this temperate glacier, consistent with previous studies examining surface energy balance controls on glacier mass balance.^28^^,^^29^

The spatial distribution of annual mass balance shows pronounced variability across the glacier, ranging from −3897 to +1428 mm w.e. (Figure 10A). Stakes in the accumulation zone generally exhibit less negative or positive mass balance values, while ablation zone stakes consistently show strong negative values, reflecting the typical elevation-dependent mass balance gradient observed on temperate glaciers.^30^ The spatial pattern of mass balance-albedo correlations reveals significant heterogeneity across the glacier surface (Figure 10B), with correlation coefficients ranging from −0.5 to +1.0. Notable clustering of positive correlations in the upper glacier regions and predominantly negative correlations in the lower ablation zones suggests that albedo-mass balance relationships are strongly influenced by elevation-dependent processes and local topographic effects, similar to patterns observed on other mountain glaciers.^31^

The glacier-wide temporal analysis reveals contrasting trends between mass balance and albedo over the study period (Figure 10C). Annual mass balance shows high interannual variability, with particularly negative values in 2016 and 2022, while maintaining a general declining trend consistent with regional glacier mass loss in Central Asia.^8^ Conversely, mean annual albedo exhibits less pronounced temporal variations, fluctuating between approximately 0.35–0.50, with values typical for temperate glacier ice and firn surfaces.^10^ The temporal decoupling between mass balance and albedo trends suggests that while albedo influences surface energy balance, other meteorological factors may dominate the overall mass balance signal at the glacier-wide scale.^32^

At the individual stake level, the overall correlation between annual mass balance and albedo is weak but statistically nonsignificant (r = −0.075, *p* = 0.191) (Figure 10D), masking substantial spatial heterogeneity in local correlations. Individual stake correlations show a broad range of relationships, with several stakes exhibiting statistically significant correlations (Figure 10E). Notably, stakes E-H3 (*p* < 0.05) and E-G3 (*p* < 0.01) demonstrate significant positive correlations, indicating that higher albedo corresponds to less negative mass balance at these locations. This spatial pattern of correlations provides insights into dominant physical processes, where positive correlations in upper glacier regions likely reflect the albedo-temperature feedback mechanism,^33^ while negative correlations in some lower elevation areas may indicate complex interactions between surface conditions and meteorological forcing.^34^

Our analysis of climatic controls on surface albedo and mass balance of UG1 over 2014–2024 provides crucial insights into glacier-climate interactions in Central Asia’s changing environment. Air temperature and snowfall are the dominant drivers of albedo variation, jointly explaining 34.6% of total variance through linear modeling, while GAM improved explanatory power to 45.7% by capturing non-linear relationships and threshold effects. AOD contributes minimally (0.1% improvement), indicating secondary importance compared to meteorological forcing. UG1 exhibits significant declining albedo (−0.006 per year) concurrent with sustained mass loss (−0.019 m w.e. per year).

Integration of high-resolution Landsat data with 45 mass balance stakes reveals pronounced spatial variability in albedo-mass balance correlations (−0.5 to +1.0). While the overall glacier-wide correlation is weak (r = −0.075, *p* = 0.191), positive correlations in upper regions reflect albedo-temperature feedback mechanisms, with individual stakes showing statistically significant relationships. Substantial seasonal variation in explanatory power (7.7%–37.7%) emphasizes complex interplay between climate variables throughout the annual cycle. GAM’s superior performance highlights the importance of non-linear modeling approaches for capturing threshold behaviors in glacier albedo processes.

### Limitations of the study

A key limitation is the lack of continuous *in situ* albedo measurements on UG1 (e.g., fixed radiometers); aside from a few handheld spot checks, we cannot rigorously validate the satellite-derived albedo time series, so retrieval biases and residual BRDF/topographic effects may persist. Landsat’s 30-m resolution, 16-day revisit, and cloud/snow masking introduce temporal gaps and mixed-pixel risks despite the 60-m inward buffer. ERA5-Land (∼9 km) and the DXG station may not resolve glacier-scale microclimates or align with satellite footprints. MCD19A2 AOD constrains atmospheric loading but not surface impurity deposition or darkening. Stake mass-balance data are spatially sparse and sometimes temporally offset from satellite overpasses, limiting pixel–stake comparability. The 2014–2024 record is short for robust attribution, and subtle Landsat-8/9 cross-sensor differences may remain. Finally, our MLR/GAM frameworks omit processes (wind redistribution, snow metamorphism, debris/biological darkening), leaving ∼54% of albedo variance unexplained and limiting generalization beyond UG1 without further *in situ* validation and full energy-balance modeling.

## Resource availability

### Lead contact

Requests for further information and resources should be directed to the lead contact, Ming Jing (mingjing@beaconscience.org).

### Materials availability

No new unique materials were generated in this study.

### Data and code availability

All codes and computed raw data are publicly available at https://github.com/petermingjing/urumqi_glacier_no1, in which (1) for the raw albedo and meteorological data, refer to the .csv files, (2) for the computing codes, refer to the .txt files, and (3) for the other items, refer to the .md and LICENSE files.

## Acknowledgments

This research is funded by the Science and Technology Project of TPRI (grant no. 092317-102), the Science and Technology Program of Gansu Province (grant nos. 23ZDFA017 and 22ZD6FA005), and the China Railway Group Limited Science and Technology Research and Development Plan (Academy of Scientific Research 2023-Major-01). The authors declare no competing interests.

## Author contributions

H.Y., W.F., Z.C., P.R., D.Z., and M.J. contributed to conceive this study; W.F. required funding; H.Y., Z.C., P.R., D.Z., and M.J. drafted the manuscript; W.F. and M.J. supervised and reviewed the manuscript from first to the final submission.

## Declaration of interests

M.J. is affiliated with a private consulting service, Beacon Science & Consulting (ABN 28737731238), registered with Australia Securities and Investments Commission (ASIC).

## STAR★Methods

### Key resources table

**Table:** 

REAGENT or RESOURCE	SOURCE	IDENTIFIER
**Deposited data**		

Albedo and climate data of UG1	Google Earth Engine Data	https://developers.google.com/earth-engine/datasets/catalog

**Software and algorithms**		

Google Earth Engine	*Google*	https://code.earthengine.google.com/
Excel 365	*Microsoft Corporation*	https://www.microsoft.com
RStudio (v2005.09.1 + 401)	*Posit Corporation*	https://posit.co/
R language (v4.5.1) and the ‘mgcv’ (v1.9) package for GAM model	*The R Foundation*	http://www.r-project.org/

### Method details

#### Data and methods

Our data collections include *in situ* mass-balance and meteorological measurements and remote sensing datasets. They are Landsat-8/9 albedo (2014–2024) for spatial/temporal analysis; glacier-wide mass balance (2000–2024) and stake records (2013–2023) for context and coupling; ERA5-Land temperature/precipitation (2000–2024) as climatic drivers; and MODIS (2000–2024) for cross-sensor context/validation, respectively.

#### Study glacier

UG1 (43°06′N, 86°49′E, 4848 m a.s.l., Figure 1) located in the eastern Tianshan Mountains of Central Asia, is one of the most intensively studied glaciers in China. It lies at the headwaters of the Urumqi River, which plays a crucial role in water supply for the arid regions downstream. UG1 consists of two branches, the East Branch and the West Branch split in 1993, with a total area of 1.59 km^2^ as of 2012, having experienced significant reductions in size and thickness due to climate change. The glacier’s elevation ranges from 3,750 to 4,480 m, and its average ice thickness was measured at 44.5 m in 2012, though it had been thinning by 0.34 m per year between 1981 and 2012.^35^

The region’s continental climate is characterized by below-freezing average annual temperatures, with summer temperatures driving glacial melt. Precipitation, mainly in the form of snow, contributes to the glacier’s mass during winter, but rising summer temperatures have significantly accelerated melt in recent decades.^36^ UG1 is an important site for glacier monitoring and has been a reference glacier for the World Glacier Monitoring Service (WGMS) since 1959. It provides crucial long-term data on mass balance, glacier terminus change, and runoff, making it a key resource for understanding glacier-climate interactions in Central Asia.

#### Ground measured albedo and mass balance data of UG1

While UG1 lacks continuous ground-based radiation or fixed albedo records, sporadic handheld measurements conducted in 2017 were compared with MODIS albedo (MCD11). The comparison showed strong agreement (R^2^ = 0.93, RMSE = 0.05, *N* = 13), providing confidence in the reliability of our satellite-derived albedo dataset, referring to Figure2B in Yue et al.^37^ Mass balance data for UG1 were derived from a combination of standardized datasets and *in situ* observations. Glacier-wide annual mass balance estimates were obtained from the World Glacier Monitoring Service (WGMS), which compiles long-term records based on the glaciological method. These data, accessible via https://wgms.ch/products_ref_glaciers/urumqi/, originate from the Tianshan Glaciological Station of the Chinese Academy of Sciences and span a continuous monitoring period since the late 1950s. In parallel, we conducted annual on-site stake measurements at UG1 to quantify surface accumulation and ablation directly. This dual-source approach enhances the spatial resolution and reliability of the mass balance record, while ensuring comparability with global glacier monitoring efforts.

#### Computing platform and dataset

We processed data using Google Earth Engine (GEE)^38^ to create time series of albedo, temperature, and precipitation for UG1. Albedo was derived from Landsat-8 OLI (30-m resolution, 16-day revisit), offering detailed surface reflectance data, with a 60-m buffer applied within glacier outlines from the Randolph Glacier Inventory (RGI) 7.0 to reduce debris effects.^39^ Daily temperature, precipitation, and snowfall data came from the ERA5-Land Daily Aggregated datasets (9-km resolution).^40^ Aerosol optical depth (AOD) data were also derived from GEE’s implanted MCD19A2 dataset.^41^ Landsat-derived albedo, validated against field measurements,^13^ outperforms coarser MODIS products for small glaciers like UG1 (below Figure), while ERA5-Land data were aligned spatiotemporally with albedo for consistent analysis. Below Figure confirms that MODIS pixels are too coarse to accurately capture albedo variability at the glacier scale, leading to our selection of Landsat-8 and -9 datasets. To minimize contamination from mixed pixels containing both glacier ice and surrounding debris, we applied a conservative 60-meter inward buffer from UG1’s boundaries for all albedo computations.

**Figure:**
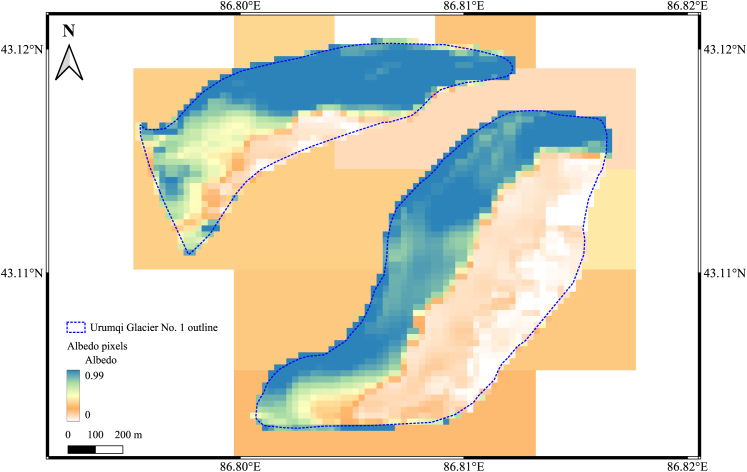
Outline of UG1 overlaid with MODIS (MOD10A1, 500 m resolution) and Landsat-8 (Collection 2, Level-2, 30 m resolution) pixels The figure shows the annual mean albedo values for the year 2023.

#### Air temperature and precipitation data: On-site vs. ERA5-land

The Daxigou (DXG) Meteorological Station (43.06°N, 86.5°E, 3543.8 m a.s.l.) locates at the UG1’s terminals and has been operating since 1958 with daily surface air temperature and precipitation data collections.^42^ We compared the monthly mean air temperature and monthly precipitation sum between ERA5-Land and DXG records. The very close relationships between precipitation and air temperature by ERA5-Land and DXG records suggests that ERA5-Land dataset can represent air temperature and precipitation over the UG1 area (Figure 2). Given the high correlations we observed (R^2^ = 0.99 for air temperature, and 0.95 for precipitation), we believe this comparison strengthens confidence in using ERA5-Land as the primary climatic input.

### Quantification and statistical analysis

To quantify the influence of climatic drivers on glacier albedo, we employed both multiple linear regression (MLR) (Figure 7) and Generalized Additive Models (GAM)^43^ (Figure 8). MLR provides a straightforward framework for assessing the linear contribution of predictors such as air temperature, snowfall amount, and aerosol optical depth (AOD), with coefficients indicating effect direction and magnitude. However, glacier–climate interactions are often nonlinear and involve threshold behaviors (e.g., temperature transitions near 0°C). To address this, we used GAMs implemented in the R package ‘mgcv’ (version 1.9), applying thin plate regression splines with smoothing parameters estimated by restricted maximum likelihood (REML). GAMs allow flexible nonlinear fits while avoiding over-parameterization, and model skill was evaluated with cross-validation, R^2^, and RMSE (Figure 9). This dual approach enabled direct comparison between linear and nonlinear formulations, testing whether GAMs improve explanatory power and capture threshold effects in albedo–climate relationships at UG1.
